# Implementation of a Clinical Pharmacist in a Hemodialysis Facility: A Quality Improvement Report

**DOI:** 10.1016/j.xkme.2020.11.015

**Published:** 2021-02-10

**Authors:** Chantale Daifi, Brian Feldpausch, Pia-Allison Roa, Jerry Yee

**Affiliations:** 1 Division of Community Care Services, Department of Pharmacy, Henry Ford Health System, Detroit, MI; 2 Division of Nephrology and Hypertension, Henry Ford Hospital, Detroit, MI

**Keywords:** Medication reconciliation, MRPs, medication-related problems, ECA, estimated cost avoidance, EMR, electronic medical record, HD, hemodialysis, MTM, medication therapy management

## Abstract

**Rationale & Objective:**

Hemodialysis (HD) patients have complicated disease states, placing them at higher risk for medication-related problems, medication discrepancies, and nonadherence. The objective of this study is to evaluate the impact of a clinical pharmacist in a single HD facility by assessing the efficacy of medication reconciliation in HD patients and evaluating the potential impact on a single health care system.

**Study Design:**

Retrospective study.

**Setting & Participants:**

Greenfield Health Systems, a wholly owned subsidiary of Henry Ford Health System, operates 14 HD facilities throughout Southeast Michigan. The West Pavilion facility is located in Detroit, MI. Patients with end-stage kidney disease included in the study had a minimum of 4 encounters with the clinical pharmacist or pharmacy interns between August 2017 and October 2018.

**Exposure:**

A clinical pharmacist performed medication reconciliation and medication reviews with HD patients to assess medication-related problems and identify gaps in care. Interventions made by the pharmacist were prespecified through a collaborative practice agreement.

**Outcomes:**

To evaluate the impact of a clinical pharmacist in an HD facility by assessing the efficacy of medication reconciliation in HD patients and evaluating the potential impact on this health system through an estimated cost avoidance.

**Analytical Approach:**

Descriptive statistics were used to collect medication-related problems and classified based on a modified Hepler-Strand approach.

**Results:**

There were 1,403 medication-related problems, with an average of 8.96 medication-related problems per patient. Adherence was the most common medication-related problem (31%). Antihypertensive medication was the most common drug class in which the pharmacist intervened (37%), followed by vitamin D analogues and calcimimetics (29%). A projected total of US $447,355 was saved.

**Limitations:**

Retrospective analysis of observational data and descriptive statistics with the potential for residual bias and confounding.

**Conclusions:**

Pharmacists in HD facilities have a positive influence on HD patients through medication management that results in cost savings.

## Graphical abstract

**Figure:**
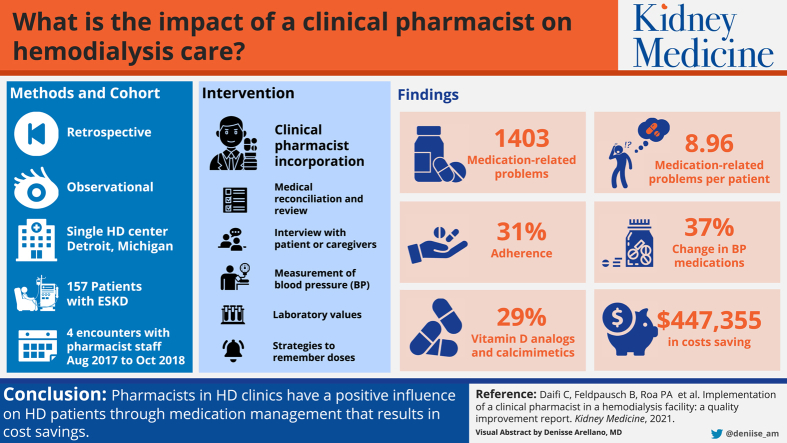

**Plain-Language Summary**

Patients with end-stage kidney disease are prescribed multiple medication regimens for management of their complex disease states. This places them at increased risk for medication-related problems. Pharmacists are trained in pharmacotherapy and can directly address medication-related problems. However, the optimal role of pharmacists in hemodialysis (HD) facilities has not been clearly defined. Prior research demonstrated that integration of a pharmacist in an HD facility provided benefits in multiple areas, including medication reconciliation and review and resolution of medication-related problems. We initiated a program in which a full-time pharmacist conducted medication reconciliation and review for each patient, with pharmacist interventions through a collaborative practice agreement. The outcomes of the program included optimization of medication management, improvement in clinical laboratory outcomes, and cost savings.

Hemodialysis (HD) patients have complicated disease states that frequently require complex medication regimens. On average, HD patients are prescribed approximately 11 to 12 medications per day, placing them at a greater risk for medication-related problems, medication record discrepancies, and nonadherence attributable in part to comorbid disease states and complex medication regimens.^1^^,^^2^ A study by Manley et al^3^ found 1,593 medication-related problems among 395 patients. A separate analysis by Alshamrani et al^4^ found 280 medication-related problems in 83 patients. The number of medication-related problems has an effect on US health care spending considering that patients with chronic kidney disease and end-stage kidney disease comprise up to 33% of Medicare expenditures.^5^

Pharmacists are specifically trained in advanced pharmacotherapy to address gaps in care and have been integrated in outpatient settings that demonstrated improvement in patient outcomes. The positive effects have stemmed from an ability to assess and prioritize high-risk patients for disease state or medication management, evaluation of patients for medication adherence, performance of basic assessments such as vital signs and point-of-care testing, ordering of laboratory tests, monitoring of the safety and efficacy of drug therapy, identification of medication-related barriers, collaboration and consultation among health care team members, and initiation, adjustment, and discontinuation of therapy.^6^

Pharmacists have increasingly been implemented as integral members of health care teams in HD facilities.^7^ The duties of the clinical pharmacist in an HD unit include interviewing patients for optimal medication reconciliation and medication review comparing the patient’s medication list against the physician’s hospital admission list, transfer, and/or discharge orders, with the goal of providing correct medications to the patient at all transition points in their outpatient care. The clinical pharmacist also provides counseling and patient education, recommendations and referrals to health care providers, delivery of a personal medication record to patients, documentation within the patient’s electronic medical record (EMR), and follow-up visits.

Although the optimal role of a clinical pharmacist in HD units is still being established, it is important to assess and evaluate the specific impact of pharmacy services on direct patient outcomes and cost savings. Several studies have documented improved blood pressure (BP) management, reduced anemia in chronic kidney disease, and improvement in calcium and phosphate levels. The latter have been associated with reduced mortality, number of hospitalizations, and inpatient length of stay and enhanced cost savings with clinical pharmacist interventions.^8^, ^9^, ^10^, ^11^, ^12^, ^13^ Although these studies show promising outcomes with pharmacy service intervention, it is important to continually assess patient outcomes and cost savings using different patient care models.

The primary objective of the study is to evaluate the impact of a clinical pharmacist in an HD facility by assessing the efficacy of medication reconciliation and review in HD patients. The secondary objective is to evaluate the potential impact on the health care system.

## Methods

### Study Design

This is a retrospective observational descriptive study that identified HD patients from Greenfield Health System (GHS), a dialysis organization that provides dialysis care for patients with end-stage kidney disease. GHS operates 14 HD facilities in Southeast Michigan, serving approximately 2,000 patients and providing all forms of kidney replacement therapy. GHS is a wholly owned subsidiary of Henry Ford Health System. West Pavilion In-Center HD Unit, a facility in Detroit, has 46 dialysis chairs and accommodates up to 268 patients. All in-center HD adult patients 18 years or older, both men and women, receiving in-center HD for a diagnosis of end-stage kidney disease between August 2017 and October 2018 were included. Exclusion criteria included patients who were minors (aged <18 years), resided in a nursing home, or received home HD, peritoneal dialysis, or dialysis for acute kidney injury. The Henry Ford Health System Institutional Review Board approved this study (Institutional Review Board #13159).

### Intervention

The dialysis facility consists of an interprofessional team that includes dieticians, physician assistants, nurse practitioners, nephrologists, and social workers. A clinical pharmacist was added to the interprofessional team in 2017 as an approach to incorporate a pharmacist-driven medication reconciliation and review service. Through a collaborative practice agreement, the pharmacist was able to order medications and laboratory tests under the scope of dialysis-related problems. This included managing BP and chronic kidney disease–mineral and bone disorder. Incorporation of clinical pharmacy services in this manner had not existed at this facility before this study.

The clinical pharmacist performed daily medication reconciliation and medication reviews with each HD patient. Medication reconciliation consisted of the patient or caregiver bringing in the patient’s home medications so the pharmacist could accurately identify the medication by looking at the name and strength. Then the pharmacist would conduct a 1-on-1 interview with the patient and/or caregivers that consisted of inquiring how the patient was taking each medication to identify whether they were taking it differently from what is directed on the bottle and if there were any problems the patient was having with their medications, such as side effects, cost, or tolerability.

After the medication reconciliation was performed, the pharmacist conducted a medication review to assess whether there were gaps in patients care, such as any medication-related problems defined by 9 categories: adherence, adverse drug reaction, dose too high, dose too low, needs additional drug therapy, unnecessary drug therapy, drug-drug interaction, wrong drug, and cost/accessibility/refills.^8^ A medication-related problem was defined as an issue related to one of those categories. The pharmacist used the collaborative practice agreement to prescribe or change dialysis and/or BP medications that were needed under the delegation of a nephrologist. Referrals to physicians were made if care outside of the collaborative practice agreement boundaries was required.

Some patients required caregiver involvement to reinforce education and medication changes. For patients requiring caregiver involvement, the caregiver was contacted with patient consent. The pharmacist attempted, when feasible, to have the caregiver present during medication reconciliation. If not possible, the caregiver would be contacted after the pharmacist conducted the visit with the patient to provide accuracy and updates regarding the medication regimen.

The findings and changes were documented in the EMR. This facility operates using an outpatient dialysis EMR (TIME) separate from the hospital EMR (EPIC). Non-nephrology health care providers use the hospital EMR and do not have access to the outpatient dialysis EMR. To circumvent this software gap in medication reconciliation, documentation was entered into both EMRs.

Each patient or caregiver was provided with a personalized medication reconciliation and calendar that delineated changes made to the medication regimen. Emphasis was made on the importance of appropriate medication administration at correct dosing times. Patients and caregivers were provided with strategies to remember doses. The pharmacist presented strategies that established routines for when medications should be taken, for example, on awakening or during meals and before or after dialysis, as well as how to set alarms or reminders for medication administration, and encouraged use of the medication calendar. Pocket pillboxes were given to patients who missed doses for a variety of reasons. Importantly, when physical access to medications was difficult or refills were required, the pharmacist evaluated these situations and arranged for dialysis chairside medication delivery, using the outpatient pharmacy associated with the facility.

Follow-up visits were performed a week after the initial medication reconciliation and on an as-needed basis. During the visits, the pharmacist reviewed any new laboratory values, vital signs, or changes to medications that were made from the medication reconciliation. Another discussion with the patient would occur for a full assessment of how they were tolerating the new regimen. The patient was given encouraging reinforcement and updates about the goals of therapy. Additional visits were deemed necessary if the patient had continued laboratory values that were not at goal, BP not within threshold, hospitalizations resulting in medication changes, or at patient or caregiver request.

A patient survey to measure self-reported adherence was provided to patients who received medication reconciliations and interventions after the third clinical pharmacist visit. If patients were unable to manage their own medications, adherence survey questions were deferred to the caregiver. Patients were considered adherent if they reported missing 4 or fewer doses of their medication in the last month. Patients reported on a scale of 0 to 10, with 0 being the lowest ranking and 10 being the highest ranking.

### Clinical and Laboratory Monitoring

Data were extracted through the internal EHR system. Extracted data included demographics, dialysis laboratory values’ 6-month average before pharmacist implementation with no medication reconciliation and 6 months after pharmacist medication reconciliation (phosphorus, calcium, parathyroid hormone [PTH], and vitamin D) and BP. Reference laboratory ranges were phosphorus, 2.5 to 5.5 mg/dL; calcium, 8.5 to 10.5 mg/dL; PTH, <300 pg/dL; and vitamin D, >30 ng/dL.

BP was measured during every dialysis treatment. The laboratory value criteria used for BP was ≤140/90 mm Hg defined using Eighth Joint National Committee and American College of Cardiology/American Heart Association guidelines. The 2014 guidelines were used because these were the guidelines in place when patients’ BPs were first assessed. Both systolic and diastolic thresholds had to be met for the patient to be considered at goal.

### Statistical Analysis

Continuous variables are presented as median with interquartile range and categorical variables are presented as frequency (Table 1). Medication-related problems were collected and classified as listed in Table 2. Each medication-related problem is associated with an estimated cost avoidance to the health system, defined in Table 3. Estimated cost avoidance was derived from average national health care use costs using a previously developed method and are updated annually to reflect inflation.^9^^,^^10^ Levels 3, 5, and 6 have approximate dollar amounts associated with them and were associated with an estimated cost avoidance of $241, $1,233, and $11,600, respectively. The model is based primarily on the medication-related problems and negative therapeutic outcomes that may occur in the absence of pharmaceutical care.

**Table 1:** Baseline Characteristics

	All Adults (N = 157)
Age, y	63.0 [26-92]
BMI, kg/m^2^	27.3 [13.6-53.6]
Sex	
Male	76 (48%)
Female	81 (52%)
Race	
African American	124 (79%)
White	6 (4%)
Hispanic	9 (6%)
Other	18 (11%)
ESKD cause	
Hypertension	62 (39%)
Diabetes mellitus	10 (6%)
Other	85 (54%)

*Note:* Values expressed as median [interquartile range] or number (percent).

Abbreviations: BMI, body mass index; ESKD, end-stage kidney disease.

**Table 2:** Medication-Related Problems Identified

Variable	Medication-Related Problems (n = 1,407)
**Types of Medication-Related Problems**	
Adherence	439 (31.3%)
Adverse drug reaction	36 (2.6%)
Dose too high	65 (4.6%)
Dose too low	184 (13.1%)
Needs additional drug therapy	303 (21.5%)
Unnecessary drug therapy	124 (8.8%)
Wrong drug	64 (4.5%)
Additional/other medication-related problem	9 (0.6%)
Drug-drug interactions	15 (1.1%)
Cost, accessibility, refills	168 (11.9%)
**Drug Classes Associated With Medication-Related Problems**	
Phosphate binders	9%
Vitamin D analogs/calcimimetics	29%
Supplements	9%
Hypertension	37%
Diabetes	1%
Hypotension	1%
Other	14%

**Table 3:** Estimated Cost Avoidance

ECA Level	Drug Class						Total	ECA
	Phosphate Binder	Vitamin D Analogues/Calcimimetics	Supplements	HTN	DM	Other		
Level 1: improved quality of care	102	103	61	252	7	155	680	
Level 2: reduced drug product cost	6	3	—	—	—	—	9	
Level 3: avoided physician visit	7	26	2	28	2	17	82	$19,762
Level 4: avoided new prescription	57	184	48	188	1	9	487	
Level 5: avoided ED visit	6	10	—	103	—	2	121	$149,193
Level 6: avoided hospital admission	—	—	—	—	4	20	24	$278,400
Level 7: avoided life–threatening event	—	—	—	—	—	—	NA	
Total cost savings							$447,355	

*Note:* N = 1,407.

Abbreviations: DM, diabetes mellitus; ECA, estimated cost avoidance; ED, emergency department; HTN, hypertension; NA, not applicable.

## Results

**Figure 1:**
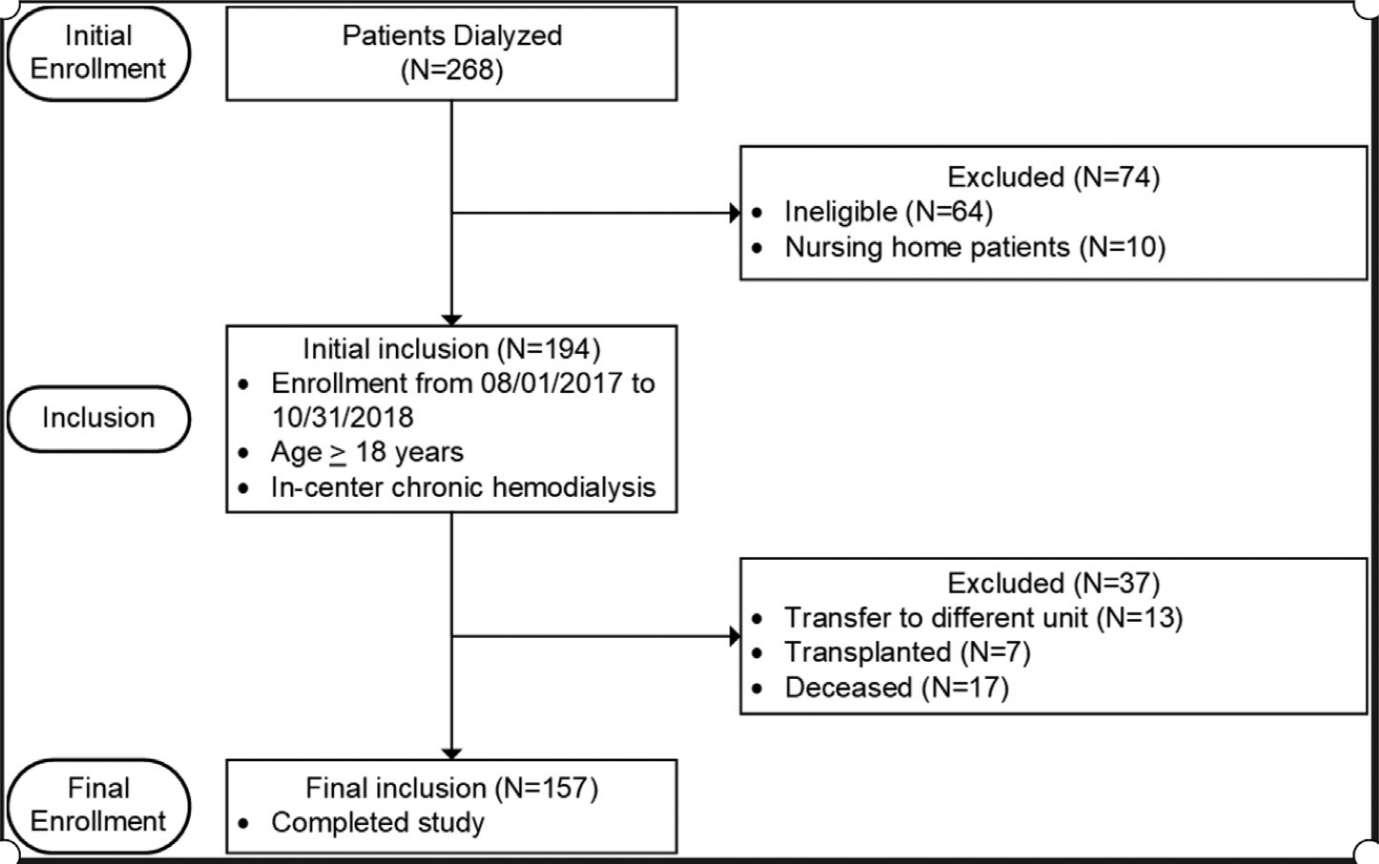
Patient enrollment.

A total of 268 patients are dialyzed at GHS West Pavilion dialysis center. Of those, 157 patients were included in the study (Fig 1). Overall, 1,407 medication-related problems were identified, with an average of 8.96 medication-related problems per patient. Adherence was found to be the most common medication-related problem (31%). The total amount of health care provider interventions made during the study period was 964, about 6.1 per patient. The most prevalent interventions were needs additional drug therapy (21.6%), dose too low (13.1%), and cost/accessibility/need for refills (11.9%). The most common drug class for which the pharmacist made interventions was medications used to treat BP (37%), followed by vitamin D analogues and calcimimetics (29%).

All medication-related problems were counted and classified to an estimated cost avoidance. It was determined that 82 interventions avoided a physician visit, 121 avoided an emergency department visit, and 24 avoided a hospital admission. Each of these had an estimated cost avoidance of $19,762, $149,193, and $278,400, respectively. A projected total of US $447,355 was saved after implementation of a clinical pharmacist in the dialysis facility.

**Figure 2:**
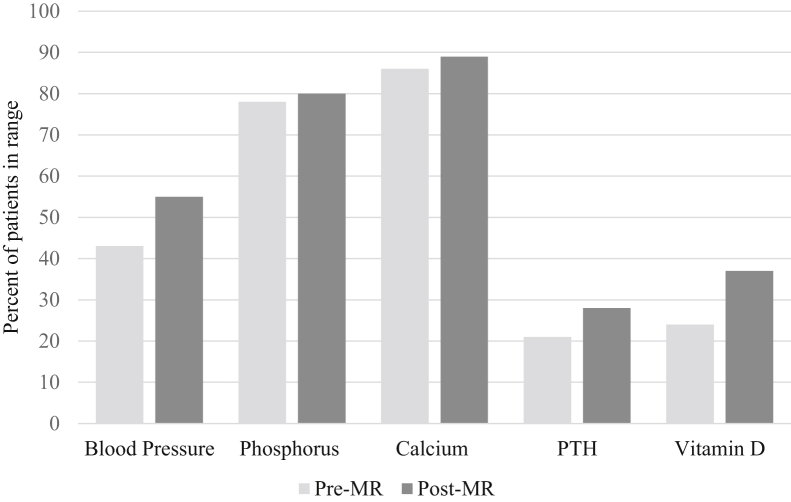
Laboratory characteristics pre– and post–pharmacist intervention. Abbreviations: MR, medication reconciliation; PTH, parathyroid hormone.

Characteristics of laboratory variables are presented in Fig 2. Phosphorus and calcium levels had an increase of 2% and 3% in the number of patients within range, respectively, and similar averages before and after medication reconciliation. Although the percent increase and averages were similar, there was still a noteworthy number of patients not within range both pre– and post–laboratory tests. A significant increase in patients in range was seen for PTH and vitamin D levels, 7% and 13%, respectively, after intervention.

Before the pharmacist intervention, 43% of patients were within the BP threshold. After medication reconciliation and pharmacist interventions, there was a 12% increase in the number of patients within the BP threshold. Also, BP target guidelines were met after the pharmacist intervention.

In the post–medication reconciliation follow-up patient survey, there were 132 respondents (Table 4). The most significant finding was that 94.7% of patients reported that the pharmacist helped them understand their medications. Furthermore, 77% of patients reported adherence to their medications after medication reconciliation.

**Table 4:** Patient Survey Results

Patient Adherence Survey		
	Answer	Results
**Q1. Do you ever forget to take your medications?**		
	Yes	88 (66.7%)
	No	44 (33.3%)
**Q1a. If yes, how many times in the past week/month would you say you have forgotten to take your medications?**		
	Adherent (≤4×/mo)	102 (77.3%)
	Nonadherent (≥5×/mo)	30 (22.7%)
**Q1b. If yes, can you please tell me why?**		
	i. Work	6 (4.5%)
	ii. Feeling sick/feeling better	6 (4.5%)
	iii. Forgetfulness	44 (33.3%)
	iv. Social activities	9 (6.8%)
	v. Not getting prescription filled on time/running out	2 (1.5%)
	vi. Cost/financial barriers	0 (0%)
	vii. Side effects	1 (0.75%)
	viii. Other (i.e. busy, tired, running late, not at home, stress)	26 (19.6%)
**Q2. How would you rate your general health?**		
	Scale 0-10	6.9
**Q3. How informed do you feel about your medications?**		
	Scale 0-10	8.3
**Q4. How comfortable are you with taking your medications?**		
	Scale 0-10	8.5
**Q5. How would you rate your current understanding of what your medications are used for and how are you supposed to use them?**		
	Scale 0-10	8.7
**Q6. Do you feel the pharmacist has helped you understand your medications?**		
	Yes	125 (94.7%)
	No	7 (5.3%)

*Note:* Values expressed as mean or number (percent).

Abbreviation: Q, question.

## Discussion

Our study demonstrates that implementation of a clinical pharmacist in an HD facility can affect patient care by resolving medication-related problems, improving clinical laboratory outcomes, and potentially reducing health care costs through the prevention of hospitalizations, with reduced lengths of stay and readmissions. The average number of medication-related problem per patient in our HD cohort was 8.96. The average number of health care interventions required in our HD patients was 6.1. A recent study demonstrated that HD patients who were discharged within 30 days from the hospital and received either partial or full medication therapy management had an average of 6.3 medication-related problems per patient.^11^

Many patients see multiple health care providers to manage their comorbid conditions or are readmitted into a hospital multiple times a year. Establishing routine follow-up with patients following initial medication reconciliation and review is helpful because medications are changed frequently during patient transitions of care. We have attributed these barriers to other factors commonly associated with this patient population, including patient education, cultural view of medications, lack of interest in health care, and financial concerns surrounding medications.

A 2-year randomized trial of patients in an HD facility in which medication therapy management services were completed by a pharmacist or standard medication reviews conducted by nurses revealed that patients who received medication therapy management had fewer hospitalizations and decreased lengths of stay compared with patients who underwent standard medication reviews by nurses.^12^ Adherence was the largest barrier identified and the most challenging to overcome given its often multifactorial origins. Motivational interviewing techniques and positive reinforcement were used to determine the root cause of nonadherence.

An investigation by Manley et al^11^ described the most common medication-related problem as that related to dosing issues (31%), that is, dose too high, dose too low, or the potential for adverse drug reaction(s). This issue was explored among a general population across 4 states and has greater generalizability than this analysis of HD patients from a single center.

In our cohort, there was an increase in the number of patients within range for PTH and vitamin D levels of 7% and 13%, respectively, and a greater proportion of patients’ BPs were within range after the intervention. We attribute the positive responses of vitamin D and BP levels to identification of adherence issues during medication reconciliation by the pharmacist and pharmacy interns. Investing in the establishment of strong patient-provider trust has the potential to augment patient health care interest and a proactive stance toward one’s health care. Thus, pharmacists not only have proximity to maintenance HD patients but also the potential to facilitate these positive outcomes.

We categorized our medication-related problems into 3 estimated cost avoidance categories and calculated cost savings of US $447,355 during the 6-month period of observation. The analysis by Ernst and Grizzle,^13^ using a model of drug-related morbidity and mortality to estimate health care costs associated with unresolved or unrecognized medication-related problems in the United States ambulatory care population, estimated that a total of US $76.6 billion was lost due to medication-related problems. The largest component of this cost was attributed to medication-related hospitalizations.^9^ The investigators updated costs 6 years later, and medication problem–related costs had more than doubled to US $177.4 billion.^13^ The medication therapy management administrative services company adopted the estimated cost avoidance model in an effort to delineate the value of pharmacists to the US health care system.^10^ The 7-year study conducted in a community setting of pharmacists who provided medication therapy management to patients determined a mean estimated cost avoidance of US $93.78 per medication therapy management service on a single patient.^10^

There are limitations of our analysis, which was retrospective with observational data and descriptive statistics. Residual bias is present because a pharmacist and pharmacy interns conducted the study. However, objective methods were used as feasible during the collection of medication-related problems to mitigate confounding. Also, classification of medication-related problem severity and quantification of cost through estimated cost avoidance levels bears a degree of subjectivity. This frequently used method was implemented to yield the most accurate representation; however, it is difficult to determine exact costs. Only 227 of 1,407 medication-related problems were associated with a dollar amount in our total estimated cost savings. Most of the 1,176 medication-related problems are valuable in that they improved patient care and cost savings but do not have an associated dollar amount.

Pharmacists can decrease medication-related problems during patients’ transitions of care among different providers. The integration of pharmacists into an interprofessional team in HD facilities potentially improves patient outcomes of BP control and increasing vitamin D levels while reducing health care costs. Overall, this study demonstrates the benefits of a dedicated HD facility pharmacist. The close relationship of the pharmacist to patients and health care providers optimizes medication management with cost savings through the provision of appropriate information regarding medications, establishment of trust, and periodic follow-up.
